# Human leukocyte antigens: the unique expression in trophoblasts and their crosstalk with local immune cells

**DOI:** 10.7150/ijbs.73616

**Published:** 2022-06-13

**Authors:** Xin-Xiu Lin, Ying-Ming Xie, Si-Jia Zhao, Chun-Yan Liu, Gil Mor, Ai-Hua Liao

**Affiliations:** 1 Institute of Reproductive Health, Center for Reproductive Medicine, Tongji Medical College, Huazhong University of Science and Technology, Wuhan, P.R. China.; 2 Department of Obstetrics, Maternity and Child health care hospital Hubei, Wuhan, PR China.; 3 C.S. Mott Center for Human Growth and Development, School of Medicine, Wayne State University, Detroit, MI, USA.

**Keywords:** pregnancy, cytotrophoblast, syncytiotrophoblast, extravillous trophoblast, HLA-C, HLA-E, HLA-F, HLA-G

## Abstract

Trophoblasts differentiate and form the placenta during pregnancy in a complex and finely orchestrated process, which is dependent on the establishment of maternal-fetal immune tolerance and the proper function of trophoblasts. Trophoblasts express HLA-C and non-classical HLA-Ib molecules (HLA-E, HLA-F, and HLA-G). Numerous studies have shown that the unique expression pattern of the HLA molecules is closely linked to the successful acceptance of allogeneic fetus by the mother during pregnancy. However, some controversies still exist concerning the exact expression and recognition patterns of HLA molecules in different trophoblast subpopulations and cell lines. Thus, we summarize three types of trophoblast subpopulations as well as the common trophoblast lineages. Then, the classification and structural characteristics of HLA molecules were elucidated. Finally, the presence of HLA-C and non-classical HLA-Ib molecules (HLA-E, HLA-F, and HLA-G) in various trophoblasts and cell lines, as well as their potential role in establishing and maintaining normal pregnancy were also discussed. Together, this review will help people comprehensively understand the complex immune interactions between maternal and fetal crosstalk during pregnancy and ultimately better understand the physiological and pathological etiologies of pregnancy.

## Introduction

In 1953, Sir Peter Medawar 1 defined the immunological paradox in pregnancy, whereby the mother would not reject a fetus carrying the father's antigen. The placenta is the interface between the mother and the fetus that separates them anatomically. Nevertheless, trophoblasts, special fetal epithelial cells, have close contact with the maternal immune system. Although it is still a mystery why the maternal immune system does not reject the allogeneic antigens, a preferred immunologic theory has been well recognized, that is the establishment of immune tolerance at the maternal-fetal interface accounts for not attacking the fetus by the maternal immune system 2.

Recent studies have shown that the immunological tolerance state maintained by the maternal immune system during pregnancy is mainly due to the specific human leukocyte antigen (HLA) expression pattern in trophoblasts 3, 4. These molecules mediate the contact between trophoblasts and maternal immune cells via their receptors. It is well-known that trophoblasts do not express HLA-II class molecules, which are surface markers of strongly immunogenic cells in allograft transplantation 5. Consequently, the absence of expression of the relevant HLA molecules in trophoblasts that cause allograft rejection may play a key role in long-term fetal survival 6. Abnormal HLA expression patterns in trophoblasts will lead to pregnancy-related diseases including recurrent miscarriage, preterm delivery, fetal growth restriction, pre-eclampsia, etc. 7.

In this review, we mainly aim to discuss the exact expression pattern of HLA molecules in trophoblasts, their interaction with local immune cells as well as the roles in establishing immune tolerance during normal pregnancy.

## Trophoblast types in human placenta

### Trophoblasts

A zygote is generated when sperm and egg interact. On the fifth day after fertilization, a preimplantation embryo is created after recurrent cleavage. The inner cell mass eventually develops into the fetus, while the outer trophectoderm proliferates and develops into the placenta. On days 7-9 after fertilization, the outer trophectoderm differentiates into cytotrophoblasts (CTBs), and a deeper, non-proliferative, invasive, multinucleated, thickened mass of primitive syncytium 8. The primitive syncytium appears to secrete enzymes that digest and loosen the decidua surrounding it, and is present for approximately 7-9 days after initially attaches to the endometrium 8-10. CTBs are highly proliferative and served as the "stem cells" of the placenta, which can differentiate into the other two types of trophoblasts, namely syncytiotrophoblasts (STBs) and extravillous trophoblast (EVTs) 10-12. STBs are distinguished by cell fusion to be terminally differentiated and multinucleated, while EVTs are distinguished from CTBs that detach from placental villi and invade into decidua 13.

STBs are located in the outer layer of the placental villi and are in direct contact with maternal blood, forming a barrier between the mother and the fetus 14. Besides, STBs have endocrine functions, secreting human chorionic gonadotropin, placental prolactin, and pregnancy-specific glycoproteins, and can transport nutrients and oxygen to the developing fetus 15, 16. EVTs can be divided into endovascular trophoblasts (eEVTs) and interstitial trophoblast (iEVTs) 12, 13. eEVTs invade the spiral arteries and replace vascular endothelial cells, whereas iEVTs invade the decidual interstitium and eventually form the placental bed in the myometrium 13, 17. EVTs can interact with the mucosa's local immune cells, reshape spiral arteries, widen previously restricted blood vessels, and boost low-pressure blood flow to the developing placenta, thereby supplying oxygen and nutrients for the growth of the fetus 18.

### Trophoblast cell lines

To clarify the role of trophoblasts in establishing maternal-fetal immune tolerance, several human trophoblast cell lines have been set up and widely used. In 1993, Graham et al. 19 established the long-living HTR8/SVneo cell line by introducing the gene-encoding simian virus 40 large T antigen into first-trimester human trophoblasts. In 2005, Feng et al. 20 established a TEV-1 cell line, which stably expressed the human papillomavirus type 16 E6/E7 gene in primary first-trimester trophoblasts via a retroviral vector pLXSN-E6/E7. In 2009, Straszewski-Chavez et al. 21 constructed an early gestational trophoblast cell line Swan 71 by infecting early gestational trophoblasts with human telomerase reverse transcriptase. In addition, several choriocarcinoma cell lines have been established, including the BeWo, JEG3, and JAR cell lines, which are also commonly used to study the properties and functions of trophoblasts 22, 23. Nonetheless, whether these trophoblast cell lines reflect the properties of primary trophoblasts and the HLA molecule expression patterns are not fully clear and need further investigation. In recent years, a three-dimensional (3D) trophoblast organoid (TO) model 15, 24, 25 and a trophoblast stem cell (TSC) model 26 have also been established, which allows us deeply understand the secrets behind the placental formation.

## Classification and structural characteristics of HLA family

### The classification of HLA family

The major histocompatibility complex, also known as HLA, is a highly polymorphic gene complex comprising over 200 genes, whose locus is located in the 3Mbp region of the short arm of chromosome 6 27, 28. They encode cell surface glycoproteins that can be used to present and recognize self- and non-self-peptides 29. HLA molecules have been widely studied in transplantation, autoimmune, bacterial, viral infections, and tumor immunotherapy 30. In addition, the unique expression pattern of non-classical HLA in placenta appears to be the most relevant mechanism for fetus to escape the recognition of maternal immune cells 31.

Usually, HLA is classified into three types based on the function and structure, including HLA-Class I, -Class II, and -Class III molecules 32. HLA-Class I molecules can be categorized into classical HLA-Ia molecules (HLA-A, HLA-B, and HLA-C) and non-classical HLA-Ib molecules (HLA-E, HLA-F, and HLA-G) 33. HLA-Class I genes encode commonly expressed proteins participating in antigen presentation, and are ubiquitously expressed on all nucleated cells 34. HLA-Class II molecules include HLA-DR, HLA-DQ, HLA-DP, HLA-DN, HLA-DM, and HLA-DO 35, 36. HLA-class II genes encode proteins involved in antigen presentation by the so-called professional antigen-presenting cells, including dendritic cells, macrophages, B cells, and thymic epithelial cells 34, 35. HLA-Class III molecules cannot be easily classified based on known or assumed functions. HLA-class III genes encode proteins involved in complement activation, hormone synthesis, inflammation, cellular stress, extracellular matrix organization, and immunoglobulin superfamily members 34, 37.

### The structural characteristics of the HLA family

**Figure 1:**
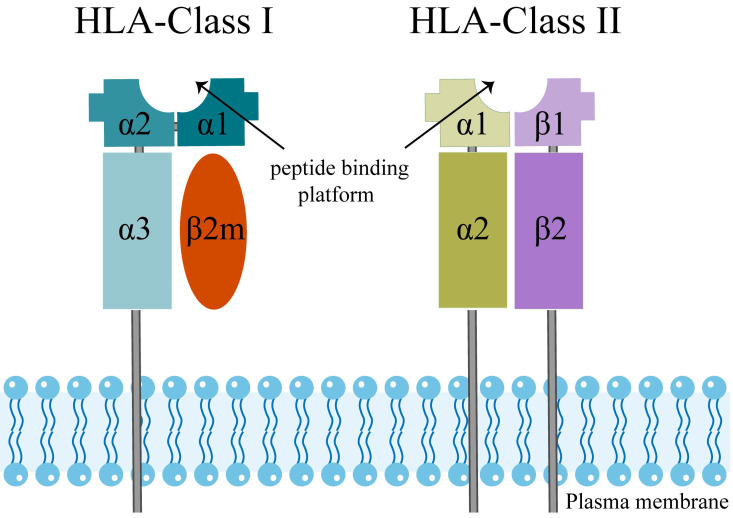
The molecular structure of human leukocyte antigen (HLA) class I and II molecules.

The extracellular structural domains of HLA-Class I molecules include heavy chain α1, α2, and α3, where α3 is non-covalently bound to light chain β2-microglobulin (β2M) 38 (Figure 1). The peptide binding platform consists of the α1 and α2 structural domains, while the α3 structural domain serves as a binding site for co-receptors 39, 40. Besides, all HLA-Class I alpha chains carry a conserved N-glycosylation site, and these glycosylation features directly correlate with the cellular localization of HLA I-like molecules 30.

HLA-Class II molecules have a similar structure to HLA-Class I (Figure 1). The two structural domains, β1 and β2 chains, evolve to form a slightly curved β-fold as a base and two α-helices at the top, which are far enough apart to accommodate a peptide chain 41. In HLA-Class II molecules, two proximal membrane immunoglobulin (Ig) structural domains support the peptide binding unit, which consists of two structural domains, being the α1 chain and the β1 chain. An Ig domain is present in each chain of HLA-Class II molecules, and a transmembrane helix anchors both chains in the membrane 39, 41. Trophoblasts do not express the HLA-Class II molecules, which are surface markers of strongly immunogenic cells in allograft transplantation 31. Therefore, we will not discuss the HLA-Class II molecules below.

## Specific expression and function of classical HLA-Ia in trophoblasts

Unlike most nucleated cells, trophoblasts do not express HLA-A and HLA-B molecules. However, trophoblasts can express polymorphic HLA-C 31, 42. Therefore, in this section, we will focus on summarizing the studies related to the expression and function of HLA-C molecules.

### HLA-C in different trophoblasts and cell lines

HLA-C was first discovered in 1970, and it is a highly polymorphic molecule 43. Although HLA-C is a classical HLA-Ia molecule, it is the only HLA-Ia class molecule expressed on trophoblasts in pregnancy. Most studies did not observe the expression of polymorphic HLA-C in CTBs and STBs by using flow cytometry 44, 45. However, other study observed that HLA-C can be weakly expressed in the cytoplasm of STBs at 5 gestational weeks and in the nucleus of CTBs at 12 gestational weeks by using immunohistochemistry method 5. Moreover, the mRNA and protein expression levels of HLA-C in placenta were significantly higher in spontaneous labor than those in non-labored C-section 5. HLA-C is expressed in all EVTs' population, including eEVTs, iEVTs, and placental bed giant cells 46. Also, it is expressed in such early pregnancy trophoblasts-derived cell lines as HTR8/SVneo, Swan 71, and TEV-1 44. While in the other cell lines, HLA-C was found to express in JEG3 and BeWo, but not in JAR 47-49. When TO differentiates into EVTs, it can express HLA-C 50.

### Function of HLA-C in pregnancy

HLA-C is the only classical HLA-Ia molecule identified on trophoblasts. HLA-C can be classified into two types based on C1 or C2 epitopes: HLA-C1 and HLA-C2 51, 52; its receptor is the Killer Immunoglobulin-like Receptor (KIR) 53. KIR is mainly expressed in NK cells, with a small proportion of metaphase T cells expressing KIR 53, 54. HLA-C1 is a ligand for inhibitory KIR2DL2 and KIR2DL3 and activating KIR2DS2, while HLA-C2 serves as a ligand for activating KIR2DS1 and KIR2DS5 as well as inhibitory KIR2DL1 55, 56. It has been found that the inhibitory role of KIR2DL1 with HLA-C2 is more critical, while the weaker inhibitory interaction between HLA-C1 and KIR2DL3 does not seem to have a significant effect on pregnancy outcome 57.

The interaction between HLA-C and inhibitory KIR in NK cells inhibits cytotoxic activity and modulates the secretion of cytokines and growth factors by NK cells, promoting EVTs' invasion and placental vascular remodeling 58. Sharkey et al 59 found that the percentage of CD56^+^ cells that expressed KIR in decidual tissues increased, and the mRNA expression levels of KIR also increased in early pregnancy. KIR2DL3, which had the highest level of KIR, led to increased binding of HLA-C1 tetramers to decidual NK (dNK) cells and would increase the production of IFN-γ, which is necessary for normal vascular remodeling and endometrial decidualization in early pregnancy 59. When the activating receptor KIR2DS1 in dNK cells binds to HLA-C on trophoblasts, it can induce dNK cells to secrete cytokines and chemokines like GM-CSF 60. The cytokines and chemokines secreted by dNK cells can induce trophoblasts to invade the decidua more effectively, promoting spiral artery remodeling and improving blood supply to the fetus 61. In addition to its involvement in immune tolerance, HLA-C can directly promote placental growth without interacting with immune cells 62. In conclusion, HLA-C molecules are critical in regulating the tolerogenic activity of NK cells in the decidua toward the fetus and trophoblast invasion.

## Specific expression and function of non-classical HLA-Ib in trophoblasts

Trophoblasts uniquely express non-classical HLA-Ib molecules, including HLA-E, HLA-F, and HLA-G 63. Therefore, this part will summarize the studies related to the expression and function of non-classical HLA-Ib molecules.

### HLA-E

#### HLA-E in different trophoblasts and cell lines

In 1988, HLA-E was identified in resting T lymphocytes 64. Moreover, in 1990, it was discovered that HLA-E was expressed in placental and extravillous tissues at all stages of pregnancy 65. The expression of HLA-E on the cell surface is regulated by the acquisition of peptides derived from the leader sequences of other HLA-I molecules, including the HLA-G and HLA-C molecules 66, 67. Hackmon et al. 5 found that HLA-E was weakly expressed in CTBs and STBs at 5 weeks of gestation and failed to detect its expression at the other gestational weeks. HLA-E was found to be expressed on the surface of EVTs 68. HLA-E was localized to iEVT in the decidual stroma by immunohistochemistry, and a small proportion of the cytoplasm of STBs also showed intense HLA-E staining 68. HTR8/SVneo, Swan 71, and TEV-1 were all found to express HLA-E 44. HLA-E was expressed on the surface of JEG3 and BeWo and was co-expressed with HLA-G 69. When TO differentiates into EVTs, it can express HLA-E 24.

#### Function of HLA-E in pregnancy

Like the classical HLA-Ia molecules, HLA-E is expressed in nearly all nucleated cells 70. HLA-E was found in 1988, and it only binds to NK cell receptors like CD94/NKG2A, CD94/NKG2B, and CD94/NKG2C, not the KIR receptor family 71-74. The binding of HLA-E to CD94/NKG2C, a receptor in CD8^+^ T cell, promotes the expansion of CD8^+^ T cell subsets and the activation of effector functions 73.

HLA-E is the major ligand of the NK cells inhibitory receptor CD94/NKG2A. Their binding can suppress NK cell-mediated cell lysis and support the fetus in evading maternal immune surveillance 72, 75. Compared with wild-type mice, NKG2A knockout mice had poor placental angiogenesis remodeling, resulting in low fetal weight and aberrant fetal brain development; both of which are the main characteristics of preeclampsia, indicating that the HLA-E/NKG2A pathway might be related to its pathogenesis 76. In addition, CD94/NKG2A is expressed in TCRγδ^+^ T cells 77. The mutual recognition between HLA-E in trophoblasts and CD94/NKG2 receptors in immune cells may be crucial in maternal-fetal immunological interactions. Further studies are required to determine whether the immune tolerance induced by the interplay of HLA-E in trophoblasts and CD94/NKG2A in decidual immune cells is the root cause of successful pregnancy.

### HLA-F

#### HLA-F in different trophoblasts and cell lines

Since less attention was paid to HLA-F, its expression and potential roles during pregnancy remain largely unknown. Based on limited literature, it is still controversial whether HLA-F is expressed intracellularly or on the cell surface. One study showed that HLA-F was expressed intracellularly in inactivated immune cells and on the surface of activated immune cells 78, while another study showed that HLA-F was expressed on the surface of EVTs 5. A study by Ishitani et al. 66 showed that low levels of HLA-F staining were observed in CTBs and STBs, but significantly higher levels of HLA-F could be detected in EVTs. Nagamatsu et al. 79 found that HLA-F was only expressed intracellularly in CTBs, STBs, and EVTs, but not on the surface of these cells. In contrary, Shobu et al. 80 discovered that HLA-F was expressed at a low level in the cytoplasm of EVTs during the first trimester but increased in the second trimester, notably in the third trimester, and also expressed on the surface of EVTs. Moreover, Ishitani et al. 66 detected the expression of HLA-F protein in several cell lines and discovered that HLA-F was not expressed on the surface of both JEG3 and Bewo cells. Other research found that HLA-F was expressed in JEG3 81. To date, no studies have been conducted to determine whether HLA-F is expressed in the trophoblast cell lines as HTR8/SVneo, Swan 71, and TEV-1.

#### Function of HLA-F in pregnancy

Although HLA-F was discovered in 1990, the related studies are still lacking 82. The heavy chain of HLA-F can form a stable complex on the cell surface by binding to β_2_m and peptide 27 or form HLA open conformers (OCs) if not bound to β_2_m and peptide 27 83. The HLA-F/β_2_m/peptide 27 complex can bind to the inhibitory immunoglobulin-like transcript (ILT) 2 receptors (LILRB1) and ILT4 (LILRB2) and may act intracellularly to regulate the expression of these inhibitory receptors 84. HLA-F OCs, a different type of HLA-F expression, can physically and functionally interact with inhibitory receptors KIR3DL1 and KIR3DL2, as well as activating receptors KIR3DS1 and KIR2DS4 83, 85, 86.

In dNK cells, 45% express the activating receptor KIR2DS4 87. HLA-F OCs can bind to KIR2DS4, so HLA-F may be involved in receptor-ligand interactions between trophoblasts and dNK cells 85. The activation of KIR2DS4 on dNK cells can secrete GM-CSF and other chemokines, which promotes trophoblast invasion 87. As a result, high levels of HLA-F expression in trophoblasts may be critical for dNK cells to promote blastocyst implantation 88. HLA-F OCs can also interact with HLA-E, indicating that HLA-F can affect the recognition of HLA-E by CD94/NKG2 heterodimers, and in turn HLA-E may alter interaction of HLA-F with KIR3DL2 85. Since HLA-F interacts with MHC class I molecules, KIR3DL2 and KIR2DS4, it is possible that HLA-F is engaged in receptor-ligand interactions between dNK cells and EVTs during pregnancy, and thus contributes to the maternal-fetal immune regulation.

### HLA-G

#### HLA-G in different trophoblasts and cell lines

HLA-G is the first HLA class I molecule found in trophoblasts. It is a non-classical HLA-Ib molecule with low polymorphism. HLA-G is composed of four membrane-bound isoforms (HLA-G1, -G2, -G3, and -G4) as well as three soluble (sHLA-G) isoforms (HLA-G5, -G6, and -G7). The roles of HLA-G in pregnancy have been extensively elucidated. HLA-G was found to localize in EVTs by using immunohistochemical staining with HLA-G monoclonal antibody (mAb) of MEM-G/9 68. During the entire pregnancy, HLA-G was found extensively expressed on the surface of EVTs by immunohistochemistry 5. Although most studies have shown that CTBs and STBs do not express membrane HLA-G, all trophoblast populations, including CTBs and STBs, can secrete sHLA-G 33, 66. For the detection of HLA-G, anti-HLA-G mAbs including 87G, 16G1, and olG were usually used 66. 87G detects both soluble and membrane-bound forms of HLA-G; 16G1 only detects the soluble form, and o1G only detects the membrane-bound form of HLA-G 33. In addition, 16G1 binding was reported to be non-specific 89.

With regards to trophoblast cell lines, Swan 71 21 and TEV-1 20 were found to express HLA-G by western blotting assay. However, other study did not detect the HLA-G expression in Swan 71 and TEV-1 by single-color flow cytometry using two different mAbs, G233 and MEMG/11 44. Therefore, different experimental methods and/or antibodies could affect the determination of the HLA-G expression on the cell surface. Besides, HTR-8/SVneo, Swan 71 and TEV-1 can express HLA-A and HLA-B molecules and express HLA-class II molecules upon IFN-γ induction 44. As early as 1990, Kovats et al. 49 discovered that HLA-G was expressed in JEG3 but not in JAR or BeWo when using W6/32 (as a mAb against pan-HLA class I molecules). On the contrary, Liu et al. 90 reported that BeWo expressed HLA-G and may be used to mimic the function of placental trophoblasts *in vitro*. However, their study did not mention which clone was used to detect HLA-G.

In addition, HLA-G gene was found to be up-regulated in the differentiation of human embryonic stem cells to the trophoblast lineage, and their expression level persisted throughout the differentiation process 91. Regarding TSCs, Sheridan et al. found they expressed HLA-A, -B, and -C but not HLA-G when using both W6/32 and MEMG-9 mAb 50. However, when TSCs were differentiated into EVTs, HLA-G expression can be detected; when TSCs were differentiated into STBs, HLA expression was downregulated 50. In contrast, TO does not express any HLA, but expresses HLA-G when differentiated to EVTs 25, 50.

#### Function of HLA-G in pregnancy

HLA-G is one of the most well-studied HLA-Ib class molecules, and its role in pregnancy has been extensively reviewed. HLA-G is the first HLA-Ib molecule identified in trophoblasts, and has a unique selective splicing pattern, limited polymorphism, and limited tissue distribution 92. HLA-G is thought to establish maternal tolerance to allogeneic fetus and could be employed as an inhibitory ligand to enhance tolerance during pregnancy 49, 93. HLA-G can promote spiral artery remodeling, immune tolerance, and fetal growth through binding to immune cells during pregnancy 94. Thus, HLA-G, also known as one of the immune checkpoint molecules, has multiple functional effects 95.

HLA-G molecules can bind to the inhibitory receptor KIR2DL4, which is expressed in dNK cells 3, mast cells 96, and some T cells 97. KIR2DL4 could inhibit NK cell-mediated cytotoxicity and induce mast cells to secrete the serine protease MMP-9 and leukemia growth factor, which promotes the invasion and differentiation of trophoblasts, and is conducive to embryo implantation 96, 98, 99. HLA-G can also inhibit cell lysis of decidual and peripheral NK cells and CD8^+^ T cells 100-102. By knocking out HLA-G on TEV-1 through RNA interference, Chen et al. 103 found that NK cells exhibited more robust killing activity against HLA-G knocked out TEV-1 when compared with the control group. The interaction between HLA-G and KIR2DL4 on the surface of dNK cells is significant for immunological tolerance.

HLA-G can also bind to the inhibitory ILT2/LILRB1 and ILT4/LILRB2 104-105, which are expressed on the surface of NK cells, CD4^+^ and CD8^+^ T cells, B cells, macrophages, and monocytes 105. ILT2/HLA-G can suppress dNK cell's cytotoxicity and increase the secretion of IFN-γ, which attributes to angiogenesis and vascular remodeling during early pregnancy 106. Furthermore, the HLA-G receptor ILT2/LILRB1 may be transferred from monocytes to activated CD4^+^ T cells and perform all functions, mediating the inhibitory effect of HLA-G on ILT2^+^ T cells 42. ILT2 and ILT4 in CD8^+^ T cells competitively bind to HLA-G and then prevent the activation of cytotoxic CD8^+^ T cells, thereby inducing the establishment of tolerance 107. Besides, when ILT2 binds to HLA-G in decidual dendritic cells (DCs), the expression of IL-10 and IL-6 by DCs increases, and the proliferation of allogeneic lymphocytes is inhibited, finally inducing the formation of tolerant DCs 108. IL-10-producing DCs drive the differentiation of initial CD4^+^ T cells into type 1 regulatory T cells via the ILT4/HLA-G signaling pathway, which is essential for maintaining tolerance to self and non-self antigens 109. ILT2 is also expressed in the decidual stroma, primarily composed of fibroblasts and macrophages, whereas ILT4 is found in the placental vascular smooth muscle 110. The receptor expression pattern suggests that HLA-G has a unique immunological function during pregnancy. The HLA-G1 homodimer connected by disulfide bonds has a higher affinity for ILT2, and the HLA-G5 free heavy chain has a more vital binding ability to ILT4 111. Differences in affinity may regulate the invasion of trophoblasts and the function of the local maternal immune response in the uterus. Recently, a unique subset of decidual memory NK cells was identified, and HLA-G induced its formation, leading to increased secretion of VEGFα and IFN-γ, thereby better supporting the critical process of placental angiogenesis 106.

## Conclusion

**Figure 2:**
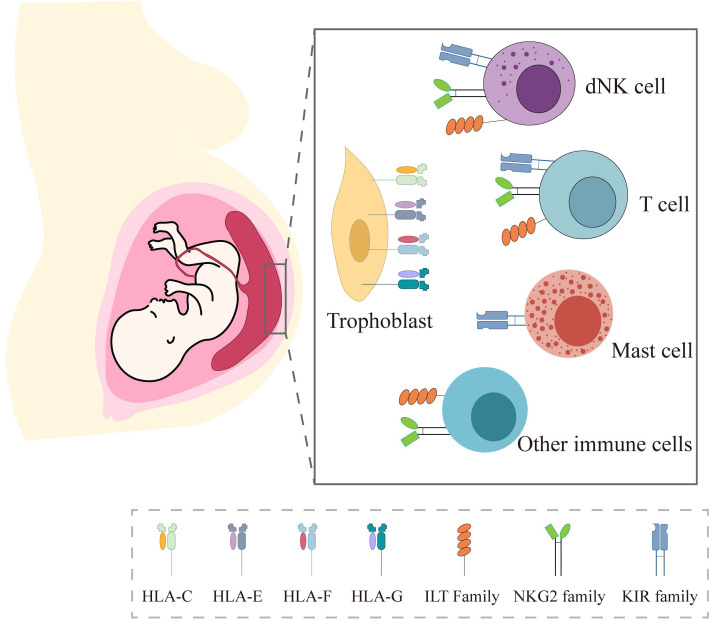
HLA expression in trophoblast and their receptors in local immune cells at the maternal-fetal interface.

Since Sir Peter Medawar first raised the immunological paradox in pregnancy, a great deal of evidence has subsequently confirmed the importance of immune tolerance underlying the mechanism of allogeneic fetus escaping the attack of maternal immune system. Among the multiple contributing factors, the unique expression of HLA molecules in trophoblasts and the interaction with their receptors in local immune cells are recognized as the key factors (Figure 2).

**Figure 3:**
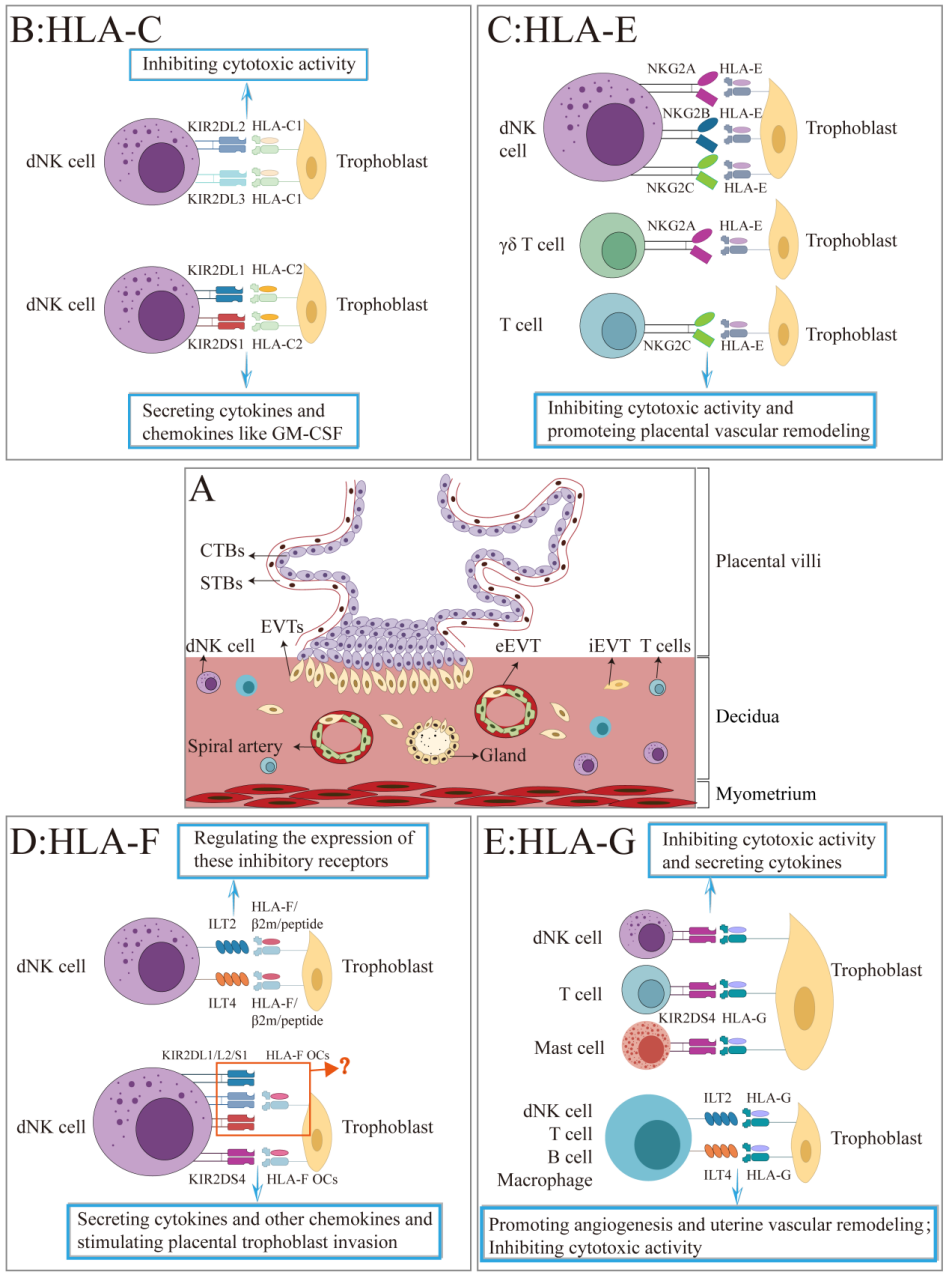
A schematic illustration of the interaction between HLA-Class I molecules and their receptors at the maternal-fetal interface. (A) The figure depicts the placental villi consisting of cytotrophoblasts (CTBs), syncytiotrophoblasts (STBs), and extravillous trophoblasts (EVTs) that invade the maternal decidua. (B-F) Summary of the interaction of HLA-C, -E, -F, and -G with their corresponding receptors on the surface of decidual NK cell, T cell, B cell, mast cell, other immune cells, and the roles during pregnancy.

In this review, we systemically described the expression pattern of HLA-class Ia molecules (HLA-C) and non-classical HLA-Ib class I molecules (HLA-E, HLA-F, and HLA-G) in three types of trophoblasts and the commonly used trophoblast cell lineages, including HTR8/SVneo, Swan 71, TEV-1, BeWo, JAR and JEG3 (Table 1). Also, we summarized how HLA-Class I in trophoblasts interact with their receptors in decidual immune cells and their roles in normal pregnancy (Figure 3). The current evidence will enhance comprehensive understanding on the complex immune mechanisms at the maternal-fetal interface.

## Figures and Tables

**Table 1:** HLA expression in primary trophoblasts and cell lines

	CTBs	STBs	EVTs	HTR8/SVneo	TEV-1	Swan 71	BeWo	JEG3	JAR
HLA-C	+/-	+/-	+	+	+	+	+	+	-
HLA-E	+/-	+/-	+	+	+	+	+	+	-
HLA-F	+/-	+/-	+	?	?	?	-	+/-	?
HLA-G	sHLA-G/-	sHLA-G/-	+	+/-	+/-	+/-	+/-	+	-
Summary	HLA-C, -E, -F, -G	HLA-C, -E, -F, -G	HLA-C, -E, -F, -G	HLA-C -E -G	HLA-C -E, -G	HLA-C -E, -G	HLA-C, -E, -G	HLA-C, -E, -F, -G	-

*Note:* +: express the HLA molecules; -: not express the HLA molecules; +/-: exist contradictory study results; ?: lack of research; CTBs: cytotrophoblasts; STBs: syncytiotrophoblasts; EVTs: extravillous trophoblasts.

## Abbreviations

CTBs: cytotrophoblasts
DC: dendritic cells
dNK: decidual NK
eEVT: endovascular trophoblasts
EVTs: extravillous trophoblasts
HLA: human leukocyte antigen
iEVT: interstitial trophoblasts
Ig: immunoglobulin
ILT: immunoglobulin-like transcript
KIR: killer immunoglobulin-like receptor
OCs: open conformers
sHLA-G: soluble HLA-G
STBs: syncytiotrophoblasts
β2m: β2-microglobulin
